# Diffusiophoresis of a Nonionic Micelle in Salt Gradients; Roles of Preferential Hydration and Salt-Induced Surfactant Aggregation

**DOI:** 10.3390/ijms232213710

**Published:** 2022-11-08

**Authors:** Eliandreina Cruz Barrios, Kyra V. Penino, Onofrio Annunziata

**Affiliations:** Department of Chemistry and Biochemistry, Texas Christian University, 2950 W. Bowie St., Sid Richardson Bldg. #438, Fort Worth, TX 76129, USA

**Keywords:** tyloxapol, magnesium sulfate, sodium sulfate, multicomponent diffusion, dynamic light scattering (DLS), polyethylene glycol (PEG)

## Abstract

Diffusiophoresis is the migration of a colloidal particle in water driven by concentration gradients of cosolutes such as salts. We have experimentally characterized the diffusiophoresis of tyloxapol micelles in the presence of MgSO_4_, a strong salting-out agent. Specifically, we determined the multicomponent-diffusion coefficients using Rayleigh interferometry, cloud points, and dynamic-light-scattering diffusion coefficients on the ternary tyloxapol–MgSO_4_–water system at 25 °C. Our experimental results show that micelle diffusiophoresis occurs from a high to a low salt concentration (positive diffusiophoresis). Moreover, our data were used to characterize the effect of salt concentration on micelle size and salt osmotic diffusion, which occurs from a high to a low surfactant concentration. Although micelle diffusiophoresis can be attributed to the preferential hydration of the polyethylene glycol surface groups, salting-out salts also promote an increase in the size of micellar aggregates, ultimately leading to phase separation at high salt concentration. This complicates diffusiophoresis description, as it is not clear how salt-induced surfactant aggregation contributes to micelle diffusiophoresis. We, therefore, developed a two-state aggregation model that successfully describes the observed effect of salt concentration on the size of tyloxapol micelles, in the case of MgSO_4_ and the previously reported case of Na_2_SO_4_. Our model was then used to theoretically evaluate the contribution of salt-induced aggregation to diffusiophoresis. Our analysis indicates that salt-induced aggregation promotes micelle diffusiophoresis from a low to a high salt concentration (negative diffusiophoresis). However, we also determined that this mechanism marginally contributes to overall diffusiophoresis, implying that preferential hydration is the main mechanism causing micelle diffusiophoresis. Our results suggest that sulfate salts may be exploited to induce the diffusiophoresis of PEG-functionalized particles such as micelles, with potential applications to microfluidics, enhanced oil recovery, and controlled-release technologies.

## 1. Introduction

The transport properties of aqueous colloidal systems are central for many technologies [1,2,3] including microfluidics [4,5,6,7,8], purification [6,7,9,10], coating [11,12], enhanced oil recovery [3,5,13,14], drug delivery [15,16,17], and detergency [17,18]. One promising way to induce the transport of colloidal particles in water is by employing directional concentration gradients of salts [8,19,20,21,22]. This transport mechanism, known as diffusiophoresis [23,24], has attracted much attention because it promotes particle focusing [8], and separation [6,7], controlled release [15], deposition [25,26], water purification [9,27], and hydrocarbon extraction [28]. Most studies on salt-induced diffusiophoresis have focused on colloidal particles and proteins that are electrically charged [6,8,9,19,20,22,26]. Here, diffusiophoresis has been described as the electrophoretic migration of a charged particle induced by the internal electric field associated with ion electrochemical gradients [8,20,24,29,30,31]. However, diffusiophoresis can also occur for neutral particles such as those coated with polyethylene glycol (PEG) motifs. Indeed, the diffusiophoresis of PEG chains [32] can be induced by employing common salting-out agents such as Na_2_SO_4_, and even osmolytes such as Trimethylamine N-oxide (TMAO). In these cases, diffusiophoresis is caused by the preferential hydration of PEG [21,33]. In other words, PEG diffusiophoresis occurs from a high to a low salt concentration in salting-out conditions due to the hydrophilicity of this macromolecule. Preferential hydration is characterized by a thermodynamic parameter, denoted as the excess of water molecules near a macromolecule compared to bulk [34,35]. It quantifies macromolecule–cosolute repulsive interactions (salting-out strength), which also encourage self-assembly processes, and is ultimately responsible for phase transitions such as segregative coacervation and crystallization [35,36].

The role of preferential hydration in diffusiophoresis can be understood by examining this transport mechanism within the framework of multicomponent diffusion [30,37,38,39]. Specifically, transport properties of a ternary macromolecule–salt–water mixture are described by a 2 × 2 diffusion coefficient matrix, in which one of the two cross-term diffusion coefficients relates to macromolecule diffusiophoresis. The other cross-term, which describes salt diffusion due to macromolecule concentration gradient, is denoted as salt osmotic diffusion [21]. It is closely related to preferential hydration and, therefore, is essential for characterizing the contribution of preferential hydration to PEG diffusiophoresis [21,40].

Diffusiophoresis can also occur in the case of supramolecular aggregates such as micelles [41,42]. These are globular particles that form by the reversible self-assembly of surfactants in an aqueous solution [43]. Due to their ability to host nonpolar molecules, micelles find applications in detergency [44], extraction [45], and catalysis [46] and as carriers for the delivery of therapeutic agents [47,48]. Thus, understanding the diffusiophoresis of micelles is interesting not only for its own sake but also because the diffusiophoresis of these carriers could be exploited for the manipulation of small guest molecules in the abovementioned applications. For instance, controlling micelle motion by diffusiophoresis is relevant to the extraction of hydrocarbons from dead-end pores [28,49], with applications in oil recovery [13] from porous rocks and soil remediation [45]. In this context, water-soluble salts such as Na_2_SO_4_ and MgSO_4_ are particularly important due to their appreciable presence in natural brines and seawater and their salting-out strength [50,51,52,53]. Indeed, both Na_2_SO_4_ and MgSO_4_ are common examples of salting-out agents, according to the Hofmeister series [36].

Recently, we have reported an experimental multicomponent-diffusion and dynamic-light-scattering (DLS) study on the ternary tyloxapol–Na_2_SO_4_–water system. The main goal of this study was to characterize the diffusiophoresis of tyloxapol micelles and its link to preferential hydration. Tyloxapol is a commercially available polyoxyethylene surfactant that is essentially an oligomer of octoxynol 9 (Triton X-100) [54,55,56]. This non-ionic surfactant forms spherical micelles with a radius of ≈3.5 nm, as determined by cryo-transmission electron microscopy [55]. Its critical micellar concentration (cmc, 0.0385 g∙dm^−3^ in water at 25 °C) is significantly lower than that of Triton X-100 (0.17 g∙dm^−3^) [55] and is predicted to further reduce in the presence of salting-out agents [57]. This implies that tyloxapol micelles are thermodynamically stable at relatively low surfactant concentrations (1–10 g∙L^−1^), with negligible free surfactant. Thus, tyloxapol micelle is a good model for globular nanoparticles that are electrically neutral, are stable in aqueous media, and have interfacial properties governed by commonly encountered PEG functionalities.

However, salting-out salts may affect micelles, not only by preferential hydration but also by enhancing surfactant aggregation [58]. Specifically, the average size of micellar aggregates can be a function of salt concentration, especially in strong salting-out conditions [41,58]. Consistent with this observation, our previous DLS experiments showed that tyloxapol micelles possess a hydrodynamic radius of ≈3.5 nm that can be approximated as a constant only at sufficiently low Na_2_SO_4_ concentrations (≲0.3 M). As Na_2_SO_4_ concentration further increases and approaches the cloud point, the hydrodynamic radius was found to significantly grow, indicating the formation of relatively large aggregates [41]. This complicates the interpretation of diffusiophoresis, as it is not clear how salt-induced aggregation contributes to micelle diffusiophoresis together with preferential hydration.

In this paper, our first objective is to determine multicomponent-diffusion coefficients for the ternary tyloxapol–MgSO_4_–water system to experimentally characterize and theoretically examine micelle diffusiophoresis for another common salting-out agent that is also geochemically relevant and possesses a stoichiometry that differs from that of Na_2_SO_4_. DLS experiments were also performed on aqueous tyloxapol solutions to characterize the effect of MgSO_4_ concentration on aggregate size. Our second objective is to examine how salt-induced aggregation affects micelle diffusiophoresis in the case of both sulfate salts. Specifically, an aggregation model explaining the observed behavior of aggregate radius as a function of salt concentration was developed and then used to theoretically evaluate its impact on micelle diffusiophoresis.

## 2. Theoretical Background

We introduce micelle diffusiophoresis within the framework of multicomponent diffusion. Specifically, we consider a ternary surfactant(1)–salt(2)–water system in isothermal conditions [19,21,29,30,59,60,61]:

(1a) $$-J_{1}=D_{11}\nabla C_{1}+D_{12}\nabla C_{2}$$

(1b) $$-J_{2}=D_{21}\nabla C_{1}+D_{22}\nabla C_{2}$$

where *J*_1_ and *J*_2_ are the fluxes of surfactant (1) and salt (2) in the volume-fixed reference frame [62], *C*_1_ and *C*_2_ are the corresponding molar concentrations, and the four *D_ij_*’s (with *i*,*j* = 1,2) are multicomponent-diffusion coefficients. The main-term coefficients, *D*_11_ and *D*_22_, describe the flux of surfactant and salt due to their own concentration gradients, while the cross-term coefficients, *D*_12_ and *D*_21_, describe the flux of a solute due to the concentration gradient of the other solute. The cross-term, *D*_12_, corresponds to salt-induced micelle diffusiophoresis, while the other cross-term, *D*_21_, describes the salt osmotic diffusion due to the surfactant concentration gradient [41].

The tyloxapol cmc is sufficiently low, such that extrapolation of thermodynamic or transport quantities at *C*_1_ = 0 yields infinite-dilution values of the micelles within experimental error [17]. Within this limit, micelle diffusiophoresis may be described by the following linear law based on non-equilibrium thermodynamics [20,37,40]:

(2) $$v_{1}=-D_{1}\left(\nabla \ln C_{1}+{\hat{D}}_{12}\;\frac{\nabla \mu_{2}}{RT}\right)$$

where *v*_1_ is the surfactant diffusion rate in the solvent-fixed reference frame [40,62,63], and *D*_1_ is the micelle tracer-diffusion coefficient. Values of *D*_1_ as a function of *C*_2_ may be obtained from DLS measurements [58,64]. In Equation (2), *µ*_2_ is the salt (2) chemical potential, with *R* and *T* being the ideal-gas constant and absolute temperature, respectively, and ∇*µ*_2_ represents the thermodynamic driving force of diffusiophoresis. This can be rewritten as ∇*µ*_2_/*RT* = *ν*_2_*y*_2_/*C*_2_, where *ν*_2_ is the number of ions in the salt formula (*ν*_2_ = 2 for MgSO_4_), and *y*_2_ is the known non-ideality thermodynamic factor of the binary salt–water system [21,50,65]. The unitless coefficient, $\hat{D}$_12_, is a reduced diffusiophoresis coefficient characterizing the magnitude of particle diffusiophoresis compared to the particle Brownian mobility, *D*_1_. Finally, the term, ∇ln*C*_1_, describes the restoring Brownian entropic force, as in the case of sedimentation in the presence of a gravitational field. The diffusiophoresis coefficient, $\hat{D}$_12_, can be obtained from the cross-term, *D*_12_, using [21]:

(3) $${\hat{D}}_{12}=\left(\frac{1}{D_{1}^{}}\underset{C_{1}\to 0}{\lim}\frac{D_{12}}{C_{1}}+\frac{{\bar{V}}_{2}}{\alpha }\right)\frac{C_{2}}{\nu_{2}y_{2}}$$

where *ν*_2_*y*_2_/*C*_2_ is the conversion factor between the salt concentration gradient, ∇*C*_2_, and the thermodynamic driving force, ∇*µ*_2_/*RT*, while $\bar{V}$_2_/*α* is a small correction accounting for the change from the volume-fixed reference frame to the solvent-fixed reference frame [40], with $\bar{V}$_2_ being the salt partial molar volume, α ≡ *D*_1_/*D*_2_ being the micelle-to-salt mobility ratio, and *D*_2_ being the salt diffusion coefficient in the solvent-fixed frame.

To describe salt osmotic diffusion, we introduce the reduced diffusiophoresis coefficient, $\hat{D}$_21_, characterizing the relative magnitude of the salt cross-term coefficient, *D*_21_, compared to the salt main-term coefficient, *D*_22_ [21]:

(4) $${\hat{D}}_{21}\equiv \underset{C_{1}\to 0}{\lim}\frac{D_{21}}{D_{22}^{}}+\alpha \frac{C_{2}{\bar{V}}_{1}}{1-C_{2}{\bar{V}}_{2}}$$

As for particle diffusiophoresis, the second term in Equation (4) is a small correction due to the reference-frame change [40], with ${\bar{V}}_{1}$ being the surfactant partial molar volume.

Salt osmotic diffusion is linked to particle–salt thermodynamic interactions, thereby providing information on the thermodynamic component of micelle diffusiophoresis [19,21,40]. Given the hypothetical limit in which particle mobility is infinitely slow compared to that of salt ions (α→0) [40]:

(5) $$\underset{\alpha \;\to \;0}{\lim}{\hat{D}}_{21}=C_{21}$$

where *C*_21_ ≡ −lim_*C*_1___→0_(*∂C*_2_/*∂C*_1_)*_µ_*_2_ is a thermodynamic coefficient [66] describing the equilibrium salt distribution along a static surfactant concentration gradient (temperature and pressure subscripts are omitted to simplify the notation). The negative sign in the definition of *C*_21_ implies that this coefficient is positive in salting-out conditions.

## 3. Results and Discussion

### 3.1. Phase Diagram

**Figure 1:**
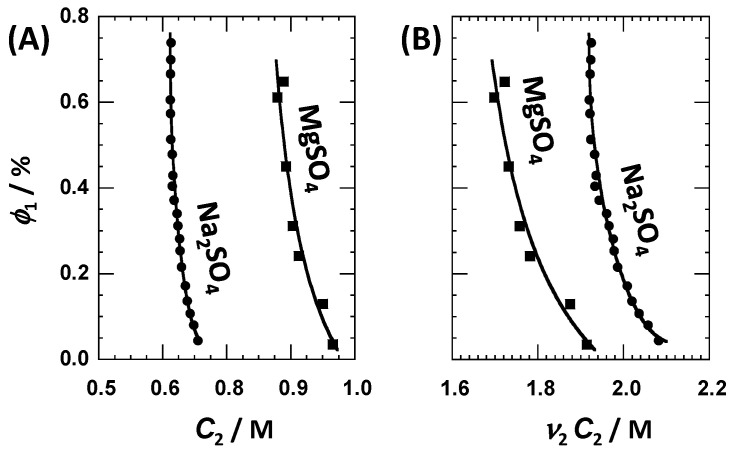
(**A**) Isothermal (*C*_2_, *ɸ*_1_) phase diagram, where *C*_2_ is salt concentration, and *ɸ*_1_ is surfactant volume fraction, showing the binodal phase boundary for the tyloxapol–MgSO_4_–water (■) and tyloxapol–Na_2_SO_4_–water (●) systems. Curves are eye guides. (**B**) Phase diagram in which salt osmolarity, *ν*_2_*C*_2_, replaces *C*_2_. Salt osmolarity needed to reach cloud point is lower in the MgSO_4_ case than in the Na_2_SO_4_ case, thereby showing that MgSO_4_ is a stronger salting-out agent than Na_2_SO_4_.

The isothermal addition of a sufficient amount of salting-out salt to an aqueous solution of polyoxyethylene surfactants leads to the separation of colloid-rich coacervates from the salt-rich phase [41,56,67]. To determine the stability domain of ternary tyloxapol–MgSO_4_–water solutions, the isothermal binodal curve (cloud points) of this system was determined at 25 °C. This phase boundary is represented in the (*C*_2_, *ɸ*_1_) phase diagram of Figure 1A, together with that previously determined for the tyloxapol–Na_2_SO_4_–water system [41]. Here, *ɸ*_1_ = *C*_1_$\bar{V}$_1_ is the tyloxapol volume fraction. Volume fractions are calculated using the known [17,68] molar volume of $\bar{V}$_1_ = 3.98 dm^3^·mol^−1^ (based on the molecular weight of 4.5 kg·mol^−1^). Within our low volume fraction range (0.02–0.7%), clouding occurs at *C*_2_ ≈ 0.9 M with MgSO_4_ and *C*_2_ ≈ 0.65 M with Na_2_SO_4_. As shown in Figure 1B, these two salt concentrations approximately correspond to the osmolarities of *ν*_2_*C*_2_ ≈ 1.8 M and ≈2.0 M, respectively. Thus, our cloud-point results indicate that MgSO_4_ is a salting-out agent that is somewhat stronger than Na_2_SO_4_, when the data are compared with respect to the total ion concentrations.

### 3.2. Multicomponent Diffusion Coefficients

Multicomponent-diffusion data are reported in Table 1. These data were obtained at the same low tyloxapol concentration of *C*_1_ = 1.00 mM (*ɸ*_1_ = 0.4%). At this composition, surfactant aqueous mixtures can be regarded as dilute micellar solutions (*ɸ*_1_ << 1).

In Table 1, the salt main-term, *D*_22_, is at least about 10-fold larger than the surfactant main-term, *D*_11_. This is consistent with micelles being significantly larger than inorganic salt ions. The surfactant main-term diffusion coefficient, *D*_11_, substantially decreases as *C*_2_ increases. At *C*_2_ = 0.65 M, *D*_11_ becomes only 33% of its value at *C*_2_ = 0. The observed decrease in *D*_11_ is large compared to the prediction based on salt viscosity alone (64%) [69]. The observed significant decrease in *D*_11_(*C*_2_) is related to a corresponding increase in osmotic compressibility as the surfactant cloud point is approached [70]. In other words, micelle concentration gradients become less effective in dissipating surfactant-rich domains in the proximity of phase separation.

The values of *D*_22_ in Table 1 are found to be just slightly lower (1.5–2.6%) than those of the binary salt–water system at the same salt concentrations (Supplementary Materials Section S1). This small difference can be attributed to a small obstruction effect [71] exerted by globular particles such as micelles on the diffusion of salt ions. At low surfactant concentration, micelles have a negligible effect on salt thermodynamic non-ideality, and *µ*_22_/*RT* = *ν*_2_*y*_2_/*C*_2_ is approximately independent of *C*_1_, even in the proximity of the binodal curve.

According to Equations (3) and (4), it is convenient to report cross-term diffusion coefficients such as *D*_12_/*C*_1_ and *D*_21_/*D*_22_. Both ratios are positive (see Table 1), implying that micelle diffusiophoresis occurs from a high to a low salt concentration, and salt osmotic diffusion occurs from a high to a low micelle concentration, respectively. At low surfactant concentration, *D*_12_/*C*_1_ and *D*_21_/*D*_22_ can be assumed [19,41] to be independent of *C*_1_, within the experimental error. Thus, they are directly used to calculate $\hat{D}$_12_ and $\hat{D}$_21_ from Equations (3) and (4). Their behavior will be discussed in Section 3.4.

### 3.3. DLS Diffusion Coefficients

**Figure 2:**
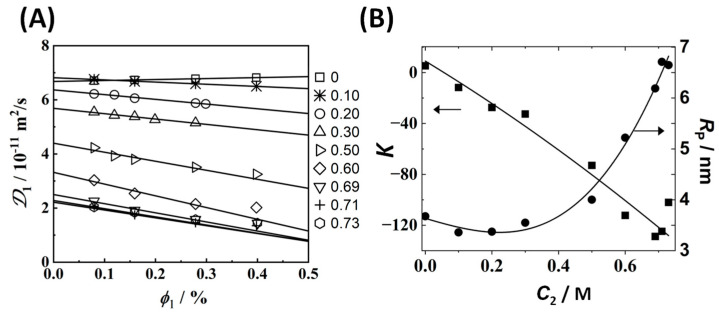
(**A**) DLS diffusion coefficient, *D*_1_, as a function of tyloxapol volume fraction, *ɸ*_1_, at several MgSO_4_ concentrations, *C*_2_/M (listed on the right) and 25 °C. Solid lines are linear fits through the data. The lines associated with *C*_2_ ≥ 0.50 M were obtained without employing the highest concentration of *ɸ*_1_ = 0.4% due to curvature. Values of 𝒟_1_ with uncertainties are reported in Supplementary Materials Section S1. (**B**) Micelle hydrodynamic radius, *R*_P_ (●), and slope, *K* (■), as a function of salt concentration. Curves are eye guides.

In Figure 2A, the DLS diffusion coefficient, 𝒟_1_ (see Supplementary Materials Section S1 for experimental values), is plotted as a function of surfactant volume fraction, *ɸ*_1_, ranging from 0.08% to 0.40% at constant salt concentrations, *C*_2_, ranging from 0 to 0.73 M, near the binodal curve. To examine our 𝒟_1_(*ɸ*_1_,*C*_2_) data, the method of least squares based on the linear relation, 𝒟_1_ = *D*_1_(1 + *Kɸ*_1_), was applied. The unitless normalized slope, *K*(*C*_2_), is known [64] to decrease as inter-micellar attractive interactions increase. At any given *C*_2_, the tracer-diffusion coefficient, *D*_1_(*C*_2_), is used to calculate the corresponding hydrodynamic radius, *R*_P_, by employing the Stokes–Einstein equation for spheres (Stokes’ radius) [64] and the known [69] viscosity of the binary salt–water systems. In Figure 2B, *R*_P_ and *K* are plotted as a function of *C*_2_. As expected for salting-out agents, *K* decreases as salt concentration increases. As in the Na_2_SO_4_ case, we identify two concentration domains from the behavior of *R*_P_(*C*_2_). For salt concentrations less than ≈0.5 M, *R*_P_ ≈ 3.5 nm is approximately constant. At salt concentrations higher than 0.5 M, *R*_P_ significantly increases, reaching the value of *R*_P_ ≈ 6.7 nm at *C*_2_ ≈ 0.7 M.

### 3.4. Micelle Diffusiophoresis and Salt Osmotic Diffusion

**Figure 3:**
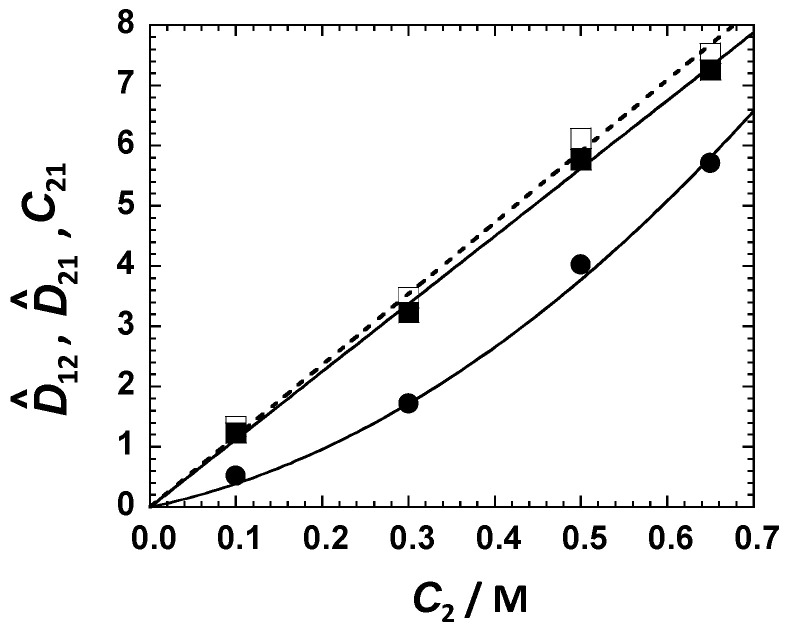
Micelle diffusiophoresis coefficient, $\hat{D}$_12_ (●), describing micelle diffusion induced by the salt concentration gradient; salt osmotic diffusion coefficient, $\hat{D}$_21_ (■), describing salt diffusion induced by the micelle concentration gradient; and preferential-interaction coefficient, *C*_21_ (□), describing salt equilibrium distribution along a micelle concentration gradient as a function of MgSO_4_ concentration, *C*_2_. Curves associated with $\hat{D}$_12_ (solid curve), $\hat{D}$_21_ (solid line), and *C*_21_ (dashed line) are fits through the data.

Cross-diffusion parameters, *D*_12_/*C*_1_ and *D*_21_/*D*_22_, in Table 1 were converted into the corresponding micelle diffusiophoresis coefficient, $\hat{D}$_12_, and salt osmotic diffusion coefficient, $\hat{D}$_21_, by employing Equations (3) and (4), respectively. Here, *D*_1_ and *α* ≡ *D*_1_/*D*_2_ were extracted from our DLS results in Figure 2 and our binary salt diffusion measurement. Values of *y*_2_ and $\bar{V}$_2_ for the binary MgSO_4_–water system were taken [50,51] from the literature (see Supplementary Materials Section S1). Our results are shown in Figure 3. Both $\hat{D}$_12_(*C*_2_) and $\hat{D}$_21_(*C*_2_) increase with *C*_2_, with $\hat{D}$_12_(0) = $\hat{D}$_21_(0) = 0, as expected [21] for neutral colloidal particles. The upward curvature in the behavior of $\hat{D}$_12_(*C*_2_) is mostly related to the significant decrease in micelle mobility *D*_1_(*C*_2_) occurring at high salt concentrations, as indicated by the behavior of *R*_P_(*C*_2_) in Figure 2B.

To theoretically examine the observed behavior of $\hat{D}$_12_(*C*_2_) and $\hat{D}$_21_(*C*_2_), it is convenient to rewrite Equation (2) in terms of thermodynamic driving forces, ∇*µ*_1_ (surfactant) and ∇*µ*_2_ (salt):

(6) $$v_{1}=-D_{1}\left(\frac{1}{m}\frac{\nabla \mu_{1}}{RT}-\lambda \;\frac{\nabla \mu_{2}}{RT}\right)$$

where *m*(*µ*_2_) is an apparent micelle aggregation number, and *λ* is a unitless Onsager transport coefficient describing the salt-induced diffusiophoresis at a constant micelle chemical potential. The negative sign preceding *λ* makes this coefficient positive in salting-out conditions [21,40]. The differentiation of *µ*_1_(*C*_1_, *µ*_2_) in Equation (6) yields:

(7) $$m\frac{\nabla \mu_{1}}{RT}=\nabla \ln C_{1}+\gamma \frac{\nabla \mu_{2}}{RT}$$

where *γ* ≡ *m* lim_*C*_1___→0_(*∂µ*_1_/*∂µ*_2_)*_C_*_1_ is another thermodynamic coefficient [66] describing the effect of salt (*µ*_2_) on the micelle chemical potential. This is thermodynamically linked to *C*_21_ (see Equation (5)) by

(8) $$\;C_{21}\;=\;(1-C_{2}{\bar{V}}_{2})\;\frac{\gamma }{m}\;+C_{2}{\tilde{V}}_{1}$$

where $\tilde{V}$_1_ ≡ $\bar{V}$_1_ − (*ν*_2_*y*_2_)^−1^$\bar{V}$_2_/*m*, with $\tilde{V}$_1_ ≈ $\bar{V}$_1_ being an excellent approximation [37,41].

The combination of Equations (6) and (7) allows us to express $\hat{D}$_12_(*C*_2_) as the difference between *γ* (*C*_2_) and *λ* (*C*_2_):

(9) $${\hat{D}}_{\;12}=\gamma -\lambda$$

The salt osmotic diffusion coefficient, $\hat{D}$_21_, is important for determining the thermodynamic and transport components in the particle diffusiophoresis coefficient, $\hat{D}$_12_. If the mobility ratio, α, is small, the preferential-interaction coefficient, *C*_21_, is approximately equal to the salt osmotic diffusion coefficient, $\hat{D}$_21_, based on Equation (5) [19,21,40]. The preferential-interaction coefficient, *γ*, can then be extracted from Equation (8), provided that *m* is known. Finally, the transport coefficient, *λ*, is calculated from the micelle diffusiophoresis coefficient, $\hat{D}$_12_, using Equation (9). More generally, we do not need to neglect *α*. Indeed, we can use the Onsager Reciprocal Relation [37,72] to show that:

(10) $${\hat{D}}_{21}=\;C_{21}-\alpha \;\frac{\lambda }{m}$$

with the second term being small compared to *C*_21_. In Figure 3, we include the values of *C*_21_ extracted from Equations (8)–(10), using the aggregation number value of *m* = 7 based on previous work [68]. Values of *C*_21_ are found to be just 3–8% larger than $\hat{D}$_21_, thereby validating that $\hat{D}$_21_ is approximately a thermodynamic quantity. Note that the chosen value of *m* is not critical for the determination of this preferential-interaction coefficient because satisfactory values of *C*_21_ can be directly obtained from *C*_21_ *≈* ${\hat{D}}_{21}$.

The value of *γ*/*m* is also approximately independent of *m* because it can be directly calculated from *C*_21_ using Equation (8), with $\tilde{V}$_1_≈ $\bar{V}$_1_. However, its interpretation is expected to generally depend on how salt affects both micelle preferential hydration and surfactant aggregation. In our case, micelle size is approximately constant for salt concentrations up to ≈0.5 M, according to Figure 2B. Thus, we may assume that micelles are colloidal particles with a fixed aggregation number within this salt concentration range. We can then describe *γ* by considering a preferential-hydration model [40,73], in which the increase in micelle chemical potential with *µ*_2_ is caused by the depletion of salt ions in the local domain of micelle (i.e., near the micelle surface). According to this model, we can write *γ*/*m* ≈ *ν*_W_$\bar{V}$_W_∙*C*_2_, where $\bar{V}$_W_ is water molar volume, and *ν*_W_ is a constant representing the number of water molecules of the local domain, in excess with respect to bulk per surfactant unit [40,41]. We extract *ν*_W_ = 450 ± 30 from our *C*_21_ data. If we assume that tyloxapol consists of ≈50 ethoxy groups, based on its chemical structure [55], we determine a thermodynamic excess of ≈9 water molecules per ethoxy group in the presence of MgSO_4_. For comparison, the value extracted for tyloxapol in the presence of Na_2_SO_4_ is ≈7 water molecules. Note that our comparison considers the difference in salt ions (*ν*_2_ = 2 for MgSO_4_ and *ν*_2_ = 3 for Na_2_SO_4_) because *γ* is defined with respect to *µ*_2_, not *C*_2_. This trend is qualitatively consistent with the cloud-point results showing that MgSO_4_ is a somewhat stronger salting-out agent than Na_2_SO_4_ (see Figure 1B).

This preferential-hydration thermodynamic model can be extended to particle diffusiophoresis by considering the presence of a slip surface boundary around the migrating particle, positioned inside the local domain of the particle [40]. It encloses the fraction of water molecules and salt ions inside the local domain that are dragged by the migrating particle (inner domain). According to this model, the ratio *λ*/*γ* is a positive constant smaller than one (inner domain fraction). Furthermore, *λ*/*γ* weakly depends on the salt salting-out strength compared to *ν*_W_. For tyloxapol in the presence of MgSO_4_, we determine *λ*/*γ* = 0.89 ± 0.03 from Equation (9), using our $\hat{D}$_12_ and *γ*/*m* data, with *m* = 7. This agrees with the value of 0.885, previously reported [41] for tyloxapol in the presence of Na_2_SO_4_.

Our analysis based on preferential hydration assumes that micelles can be treated as colloidal particles with a fixed molecular weight. However, the observed increase in micelle hydrodynamic radius, *R*_P_, at high salt concentrations (see Figure 2B) indicates that salt induces the formation of surfactant aggregates with a molecular weight larger than that of micelles in water. Here, diffusiophoresis may be related not only to preferential-hydration but also to the salt-induced change in the surfactant aggregation state. The contribution of the latter mechanism to diffusiophoresis will be examined in Section 3.5.

### 3.5. Role of Salt-Induced Surfactant Aggregation

**Figure 4:**
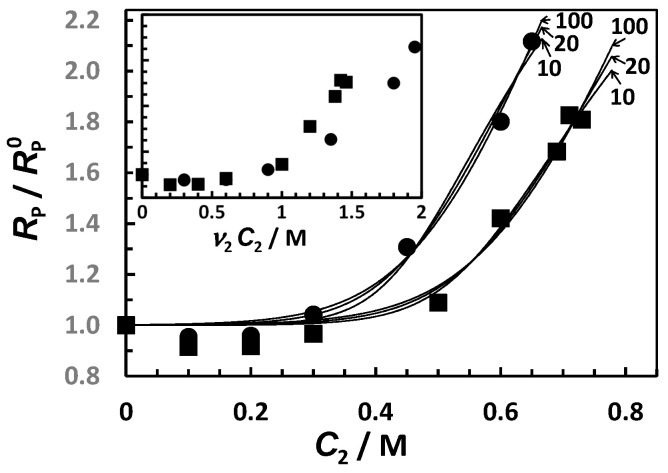
Normalized Stokes’ radius, *R*_P_/ $R_{P}^{0}$, as a function of salt concentration, *C*_2_, with $R_{P}^{0}$ being *R*_P_ at *C*_2_ = 0 (Na_2_SO_4_, ●; MgSO_4_, ■). Curves are fits through the data based on Equations (12) and (14), as discussed in the text below. Employed values of *a* (see Equation (11) for definition) are appended to each curve. Inset shows the same data plotted as a function of salt osmolarity, *ν*_2_*C*_2_.

In Figure 4, we plot the normalized behavior of Stokes’ radius, *R*_P_ (*C*_2_), for tyloxapol in the presence of MgSO_4_ and Na_2_SO_4_, with $R_{P}^{0}$ being *R*_P_ at *C*_2_ = 0. For both salts, there is a salt concentration range in which *R*_P_ is approximately constant. After salt concentrations of ≈0.3 M (for Na_2_SO_4_) and ≈0.5 M (for MgSO_4_) are reached, *R*_P_/$R_{P}^{0}$ significantly increases with *C*_2_. Note that salt-induced aggregation follows the same trend shown for the cloud points in Figure 1 (see figure inset for comparison with respect to osmolarity).

**Figure 5:**
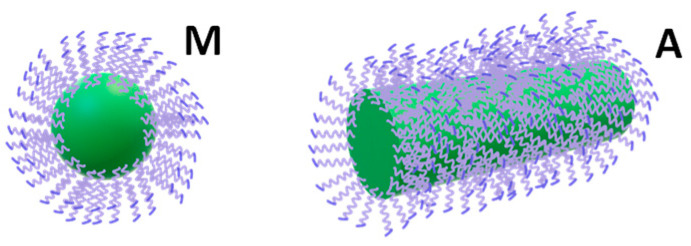
Spherical micelle (M) and worm-like aggregate (A) with the same diameter as a micelle.

The observed strong upward convexity of *R*_P_/$R_{P}^{0}$ indicates that salt is not promoting a stepwise steady growth in the aggregate size but rather a substantial cooperative change in the surfactant aggregation state. We can approximately describe this process by assuming that surfactant aggregation can occur in two distinct aggregation states in a chemical equilibrium. At low salt concentrations, spherical micelles are more stable thermodynamically. As *C*_2_ increases, micelles’ thermodynamic stability decreases due to preferential hydration. Correspondingly, a different aggregation state, which involves a relatively large number of surfactant unimers, becomes thermodynamically more favorable. In other words, relatively large aggregates can better tolerate harsh salting-out conditions than micelles. For instance, these aggregates may optimize contacts between PEG chains and reduce their exposure to salt ions by having a relatively large curvature radius compared to micelles. Furthermore, according to geometric considerations based on surfactant molecular structure [54,74], surfactant aggregates that are large compared to micelles cannot be spherical. Accordingly, an increase in micelle ellipticity occurs, which may lead to the formation of worm-like aggregates with a thickness comparable with micelle diameter, as illustrated in Figure 5. Consistent with the description, we propose that micelles (M) are in chemical equilibrium with relatively large aggregates (A). Since tyloxapol cmc is low, we shall ignore free unimers and focus on the reversible reaction *a*M ⇌ A, where *a* > 1 is the molecular-weight ratio between the aggregate and micelle. The extent of aggregation, which increases with salt concentration, can be described by introducing the fraction of surfactant in the aggregate state, *X*_A_, with 1 − *X*_A_ being the corresponding fraction in the micelle state (neglecting the small contribution of free unimers).

**Figure 6:**
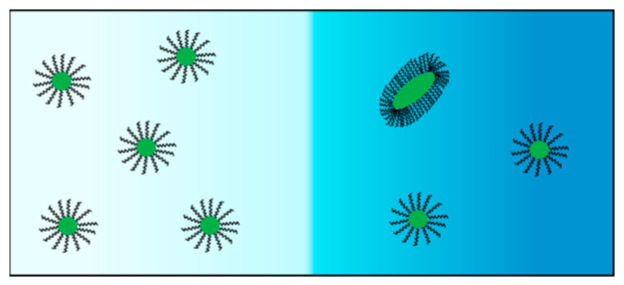
Two aqueous surfactant solutions with the same surfactant concentration but different salt concentrations are interfaced. The salt difference is portrayed as a color contrast for simplicity (left, low salt; right, high salt). The aggregate (elongated particle) depicted on the right side corresponds to the mass of three micelles. Consequently, the number of micelles on the left side (five) is higher than that on the right side (two).

From a qualitative point of view, we expect that an increase in *X*_A_ with *C*_2_ should produce diffusiophoresis from a low to a high salt concentration (negative diffusiophoresis). This effect negatively contributes to the observed value of $\hat{D}$_12_(*C*_2_). To explain this mechanism, we consider two solutions in contact with each other, with the same surfactant concentration but different salt concentrations, as illustrated in Figure 6. Since the extent of aggregation is larger in the solution at a higher salt concentration, the concentration of micelle species (M) is larger in the solution at a lower salt concentration. This causes micelle diffusiophoresis from a low to a high salt concentration. Note that the compensating difference in the aggregate (A) concentration is responsible for the aggregate diffusiophoresis in the opposite direction. However, the latter effect is relatively less important because the mobility of aggregates is low compared to that of micelles. Thus, salt-induced surfactant aggregation should produce surfactant diffusiophoresis from a low to a high salt concentration.

**Figure 7:**
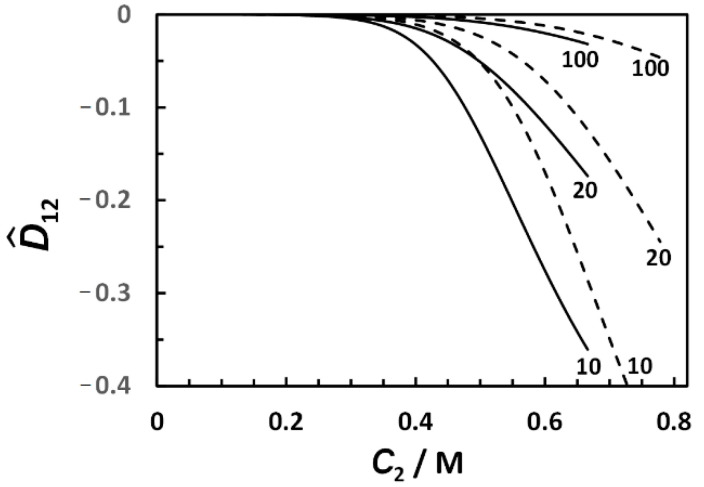
Reduced diffusiophoresis coefficient, $\hat{D}$_12_, as a function of salt concentration, *C*_2_, calculated using Equation (13) (Na_2_SO_4_, solid curves; MgSO_4_, dashed curves). Employed values of *a* are appended to each curve.

We use our two-state model together with the experimental behavior of *R*_P_/$R_{P}^{0}$ shown in Figure 4 to quantitatively evaluate the role of salt-induced aggregation on diffusiophoresis. Details on this model are in Supplementary Materials Section S2. Since the formation of aggregates becomes appreciable only at a high salt concentration, we assume that *X*_A_ << 1 at *C*_2_ = 0. The chemical-equilibrium condition between micelles and aggregates may be written in the following way:

(11) $$\ln\frac{X_{A}/a}{{\left(1-X_{A}\right)}^{a}}=\;K_{2}\;(C_{2}-C_{2}^{*})$$

where the argument in the logarithm is the equilibrium constant associated with chemical equilibrium *a*M ⇌ A. On the right side of Equation (11), *K*_2_ is a salting-out constant characterizing salt effectiveness in promoting aggregate formation, while $C_{2}^{*}$ is a critical salt concentration above which aggregates become thermodynamically favored compared to micelles. For a given set of *a*, *K*_2_, and $C_{2}^{*}$ values, Equation (11) can be numerically solved to yield *X*_A_ as a function of *C*_2_. To establish that this model is consistent with the observed increase in the Stokes’ radius, we need to derive mathematical expressions for both *R*_P_/$R_{P}^{0}$ and $\hat{D}$_12_. This is achieved in the following way (see Section S2 for more details). We first assume that individual diffusion of the micelle (M) and aggregate (A) can be described by the simple diffusion law: *J_i_* = *−D_i_*∇*C_i_*, with *i* = M and A, and *J_i_*, *D_i,_* and *C_i_* denoting the flux, diffusion coefficient, and concentration of species *i*, respectively. We then express concentration gradients, ∇*C*_M_ and ∇*C*_A_, as a function of ∇*C*_1_ and ∇*C*_2_, using Equation (11) and assuming that chemical equilibrium is fast [42] compared to diffusion. Finally, we derive the expression of the total surfactant flux from themass balance, *J*_M_ + *aJ*_A_ = *J*_1_/*m*, noting that *J*_1_ = −*C*_1_*D*_M_($R_{P}^{0}$/*R*_P_)(∇ln*C*_1_ + $\hat{D}$_12_ *ν*_2_∇ln*C*_2_) and ignoring the salt thermodynamic non-ideality. This leads to (see Supplementary Materials Section S2):

(12) $$\frac{R_{P}}{R_{P}^{0}}=\;\frac{1-X_{A}+a\;X_{A}}{1-X_{A}+a\;X_{A}\alpha_{a}}$$

and

(13) $${\hat{D}}_{12}=-\frac{(1-X_{A})X_{A}}{1-X_{A}+a\;X_{A}\alpha_{a}}\frac{(1-\alpha_{a})K_{2}C_{2}}{\nu_{2}}$$

where *α_a_* ≡ *D*_A_/*D*_M_ is a mobility ratio. Consistent with the previous qualitative analysis, $\hat{D}$_12_ < 0, if aggregates are slow compared to micelles (*α_a_* < 1). To reduce the number of parameters in our model, we assume that worm-like aggregates can be treated as prolate ellipsoids, with a minor axis equal to the micelle diameter. In this case, *α_a_* becomes the following function [75,76] of *a*:

(14) $$\alpha_{a}=\frac{\ln\left(a+\sqrt{a^{2}-1}\right)}{\sqrt{a^{2}-1}}$$

with *α_a_* ~ *a*^−1^, when *a* → ∞ (see Section S2 for more details). For comparison, *α_a_* = *a*^−1/3^ for spherical aggregates. Figure 4 shows the best fits obtained by applying the method of least squares to Equation (12), with three representative values of *a* = 10, 20, and 100, which reasonably describe the experimental behavior. We found that the observed two-fold increase in *R*_P_/$R_{P}^{0}$ shown in Figure 4 is not described well by Equation (12) if *a* < 10. This is related to the dependence of *α_a_* on *a*, which is generally weaker than *a*^−1^. The extracted values of *K*_2_ and $C_{2}^{*}$ (see Table S5 in Section S2) were then used to calculate $\hat{D}$_12_(*C*_2_) from Equation (13). As we can see from this figure, the calculated values of $\hat{D}$_12_(*C*_2_) are either small or comparable with the experimental error (5%) of the diffusiophoresis data shown in Figure 7. Thus, our analysis indicates that salt-induced aggregation plays a marginal role in surfactant diffusiophoresis compared to preferential hydration.

## 4. Experimental Section

### 4.1. Materials

Tyloxapol (BioXtra; 4.5 kg mol^−1^) and magnesium sulfate (ACROS organics, MgSO_4_; 120.37 g∙mol^−1^, purity ≥ 99.0%) were purchased from Millipore-Sigma (Burlington, MA, USA). More information on the molar mass of tyloxapol micelles and polydispersity can be found in ref. [68]. These materials were used as received, without further purification. Deionized water was passed through a four-stage Millipore filter system to provide high-purity water (0.06 µS) for all the experiments. A stock solution of MgSO_4_–water was prepared due to salt hygroscopicity. Its composition was determined from density measurements and the known density–composition relation [51]. A stock solution of tyloxapol–water was prepared by weight using a Mettler-Toledo AT400 analytical balance. Ternary tyloxapol–MgSO_4_–water solutions were obtained by combining precise masses of tyloxapol stock solution and MgSO_4_ stock solution inside flasks, and water was then added to reach the established tyloxapol and MgSO_4_ concentrations. To calculate molar concentrations, solution densities were determined at 25.00 °C, employing a Mettler-Paar DMA40 density meter, and thermostated with a well-regulated (±0.001 °C) large water bath. Tyloxapol (1) and salt (2) molar concentrations, *C*_1_ and *C*_2_, were based on the molecular weights of 4.5 kg mol^−1^ and 120.37 g∙mol^−1^, respectively.

### 4.2. Rayleigh Interferometry

Multicomponent diffusion coefficients were measured at 25.00 °C with the Gosting Diffusiometer operating in the Rayleigh interferometric optical mode [31,77,78]. In brief, an experiment starts by preparing a sharp boundary between two solutions of different solute concentrations located inside a vertical diffusion channel located inside a well-regulated water bath (±0.001 °C). The measured diffusion coefficients correspond to the average concentrations of the two interfaced solutions. Rayleigh fringes shift horizontally as the refractive index inside the diffusion channel changes along the channel vertical position, *x*. This shift is directly proportional to the refractive index, *n*(*x*). The total number of fringes, *J*, is related to the difference in refractive index between the two solutions, Δ*n*, by *J* = (*a*/*λ*)Δ*n*, where *a* = 2.5 cm is the channel width. We obtain refractive-index profiles at 50 different values of time, *t*, during the course of each experiment. The experimental refractive-index profile is then described by the normalized anti-symmetric function *f*(*y*) ≡ 2[*n*(*y*) − $\bar{n}$]/Δ*n*, where $\bar{n}$ is the average refractive index between the two solutions, *y* ≡ *x·t*^−1/2^/2, and 0 ≤ *f* ≤ 1. In our experiments, differences in concentrations between the two interfaced solutions were chosen such that *J* ≈ 50 [31]. A minimum of two experiments is required for determining the four diffusion coefficients at a given set of average concentrations. These two experiments must have different combinations of solute concentration differences across the diffusion boundary. To verify reproducibility, two other duplicate experiments are performed. To obtain *J* ≈ 50 in the experiments with a tyloxapol average concentration of 1.00 mM, gradients of tyloxapol concentration were produced by interfacing a solution at 1.77 mM with a solution at 0.23 mM. Note that both concentrations are well above tyloxapol cmc (0.009 mM). Salt concentration gradients were prepared by interfacing solutions with a salt concentration difference of ≈0.06 M (*J* ≈ 50). The four ternary diffusion coefficients in the volume-fixed reference frame, *D_ij_*, were extracted by applying a method of the non-linear least squares to *f*(*y*) data [79]. Due to tyloxapol molecular-weight polydispersity, a corrective procedure [80] was applied to our *f*(*y*) profiles to remove the contribution of tyloxapol polydispersity. This procedure is based on the *f*(*y*) profile obtained by interfacing a bottom solution with tyloxapol concentration at 1.77 mM, with a top solution at 0.23 mM in the absence of salt.

### 4.3. Dynamic Light Scattering (DLS)

DLS measurements were performed on tyloxapol–MgSO_4_–water solutions at 25.0 ± 0.1 °C. All samples were filtered using a 0.02-µm filter (Anotop 10, Whatman, Maidstone, UK) to remove dust. Experiments were carried out on a light-scattering apparatus built using the following main components: He-Ne laser (35 mW, 632.8 nm, Research Electro-Optics, Boulder, CO, USA); manual goniometer and thermostat (Photocor Instruments, College Park, MD, USA); multi-tau correlator, APD detector, and software (PD4042, Precision Detectors, Bellingham, MA, USA). All measurements were performed at a scattering angle of 90°. The scattering vector, *q* = (4π*n*/*λ*)∙sin(*θ*/2), was calculated using *λ* = 632.8 nm and the refractive index, *n*. To calculate *n*, we applied small corrections on the refractive-index value of water, 1.3314, due to MgSO_4_ concentration by using previously reported [51] refractive-index increments. The scattered-intensity correlation functions were examined employing a regularization algorithm (Precision Deconvolve 32, Precision Detectors, Bellingham, MA, USA) [21]. All normalized scattered-intensity distributions were found to be monomodal, and the corresponding *z*-average diffusion coefficient, *D*_1_, was extracted [64].

### 4.4. Cloud Point Measurements

All experiments were performed by incubating samples (≈10 cm^3^) in a well-regulated (±0.001 °C) water bath at 25.00 °C for about one hour. An exploratory set of tyloxapol–MgSO_4_–water mixtures was initially prepared by weight, with tyloxapol and MgSO_4_ concentrations varying from 0.1 to 2.0 mM and 0.05 to 1.00 M, respectively. By visual inspection, it was determined that cloud points were located within the salt concentration range of 0.85–0.95 M at all investigated surfactant concentrations. To precisely characterize cloud-point composition, a second set of ternary mixtures was prepared with a MgSO_4_ concentration fixed at 0.95 M and tyloxapol variable concentrations, again varying from 0.1 to 2.0 mM. At this salt concentration, all mixtures were observed to be cloudy. Small amounts of water (≈0.02 g) were then incrementally added to each sample. A given water addition was followed by measurement of sample total mass, stirring, and incubation at 25.00 °C. The minimum amount of solvent producing clear homogenous samples by visual inspection was chosen to identify cloud-point composition.

## 5. Conclusions

There are many examples of colloidal particles with interfacial properties governed by hydrophilic PEG chains [81,82,83]. For these particles, diffusiophoresis can be observed in the presence of the gradients of salting-out agents due to PEG hydrophilicity. We have successfully characterized the diffusiophoresis of tyloxapol micelles in the presence of a salting-out agent (MgSO_4_) and examined this transport phenomenon within the framework of multicomponent diffusion. As for the Na_2_SO_4_ case, micelle diffusiophoresis occurs from a high to a low MgSO_4_ concentration. Our multicomponent diffusion data also allowed us to characterize the salt osmotic diffusion coefficient, $\hat{D}$_21_, which is crucial for unraveling the thermodynamic and transport components of the diffusiophoresis coefficient, $\hat{D}$_12_. We applied a preferential-hydration model to the $\hat{D}$_12_(*C*_2_) and $\hat{D}$_21_(*C*_2_) data in Figure 3 and extracted two parameters describing the experimental behavior: the thermodynamic excess of water molecules in the micelle local domain, *ν*_W_ = 450 (≈8 water molecules per ethoxy group), and the inner domain fraction, *λ*/*γ* = 0.89. As in the Na_2_SO_4_ case, our DLS results show that micelle size significantly increases at high MgSO_4_ concentrations. A two-state aggregation model was, therefore, developed (Section 3.5) to describe the effect of MgSO_4_ and Na_2_SO_4_ concentrations on the Stokes’ radius of tyloxapol micelles (see Figure 4). Extracted parameters describing observed salt-induced surfactant aggregation were then used to theoretically calculate $\hat{D}$_12_(*C*_2_), ignoring the contribution of preferential hydration. The magnitude of the calculated negative values of $\hat{D}$_12_(*C*_2_) was found to be small compared to that of the corresponding experimental values, indicating that preferential hydration is the main mechanism causing micelle diffusiophoresis. We believe that the concentration gradients of salting-out agents such as MgSO_4_ and Na_2_SO_4_ may be employed for achieving the migration of PEG-based colloidal particles, such as those utilized as drug carriers and extracting agents with applications in the fields of microfluidics, enhanced-oil recovery [13], soil remediation [45], and controlled release technologies [15,16].

## Figures and Tables

**Table 1:** Ternary diffusion coefficients, *D_ij_*, at 25 °C for the tyloxapol–MgSO_4_–water system and tyloxapol molar concentration of *C*_1_ = 1.00 mM.

*C*_2_/M	*D*_11_^a^	*D*_22_	*D*_12_/*C*_1_	*D*_21_/*D*_22_
0	6.96 ± 0.01 ^b^			
0.100	6.33 ± 0.02	58.5 ± 0.1 ^b^	59 ± 1 ^c^	1.18 ± 0.15
0.300	5.14 ± 0.03	48.9 ± 0.1	50 ± 1	3.09 ± 0.06
0.500	3.51 ± 0.02	44.2 ± 0.2	54 ± 3	5.57 ± 0.06
0.650	2.33 ± 0.01	42.2 ± 0.2	39 ± 4	7.08 ± 0.05

^a^ Subscripts “1” and “2” indicate surfactant and salt, respectively. See Equation (1a,b) for definitions of *D*_11_, *D*_12_, *D*_21,_ and *D*_22_. ^b^ Values in 10^−11^ m^2^·s^−1^. ^c^ Values in 10^−11^ m^2^·s^−1^·M^−1^.

## Supplementary Materials

The following supporting information can be downloaded at: https://www.mdpi.com/article/10.3390/ijms232213710/s1.

## References

[1] Grier D.G. (2003). A revolution in optical manipulation. Nature.

[2] Ha D., Seo S., Lee K., Kim T. (2019). Dynamic transport control of colloidal particles by repeatable active switching of solute gradients. ACS Nano.

[3] Frimmel F.H., von der Kammer F., Flemming H.C. (2007). Colloidal Transport in Porous Media.

[4] Whitesides G.M. (2006). The origins and the future of microfluidics. Nature.

[5] Park S.W., Lee J., Yoon H., Shin S. (2021). Microfluidic investigation of salinity-induced oil recovery in porous media during chemical flooding. Energy Fuels.

[6] Shin S. (2020). Diffusiophoretic separation of colloids in microfluidic flows. Phys. Fluids.

[7] Dey K.K., Das S., Poyton M.F., Sengupta S., Butler P.J., Cremer P.S., Sen A. (2014). Chemotactic separation of enzymes. ACS Nano.

[8] Abecassis B., Cottin-Bizonne C., Ybert C., Ajdari A., Bocquet L. (2008). Boosting migration of large particles by solute contrasts. Nat. Mater..

[9] Shin S., Shardt O., Warren P.B., Stone H.A. (2017). Membraneless water filtration using co2. Nat. Commun..

[10] Guha R., Shang X., Zydney A.L., Velegol D., Kumar M. (2015). Diffusiophoresis contributes significantly to colloidal fouling in low salinity reverse osmosis systems. J. Membr. Sci..

[11] Sear R.P., Warren P.B. (2017). Diffusiophoresis in nonadsorbing polymer solutions: The asakura-oosawa model and stratification in drying films. Phys. Rev. E.

[12] Larson R.G. (2014). Transport and deposition patterns in drying sessile droplets. Aiche J..

[13] Liu R., Du D.J., Pu W.F., Zhang J., Fan X.B. (2018). Enhanced oil recovery potential of alkyl alcohol polyoxyethylene ether sulfonate surfactants in high-temperature and high-salinity reservoirs. Energy Fuels.

[14] Sinz D.K.N., Hanyak M., Darhuber A.A. (2013). Self-induced surfactant transport along discontinuous liquid-liquid interfaces. J. Phys. Chem. Lett..

[15] Shin S., Doan V.S., Feng J. (2019). Osmotic delivery and release of lipid-encapsulated molecules via sequential solution exchange. Phys. Rev. Appl..

[16] Wesselingh J.A. (1993). Controlling diffusion. J. Control. Release.

[17] Zhang H.X., Annunziata O. (2008). Modulation of drug transport properties by multicomponent diffusion in surfactant aqueous solutions. Langmuir.

[18] Shin S., Warren P.B., Stone H.A. (2018). Cleaning by surfactant gradients: Particulate removal from porous materials and the significance of rinsing in laundry detergency. Phys. Rev. Appl..

[19] Annunziata O., Buzatu D., Albright J.G. (2012). Protein diffusiophoresis and salt osmotic diffusion in aqueous solutions. J. Phys. Chem. B.

[20] Fahim A., Annunziata O. (2020). Amplification of salt-induced protein diffusiophoresis by varying salt from potassium to sodium to magnesium chloride in water. Langmuir.

[21] McAfee M.S., Zhang H., Annunziata O. (2014). Amplification of salt-induced polymer diffusiophoresis by increasing salting-out strength. Langmuir.

[22] Velegol D., Garg A., Guha R., Kar A., Kumar M. (2016). Origins of concentration gradients for diffusiophoresis. Soft Matter.

[23] Anderson J.L. (1989). Colloid transport by interfacial forces. Annu. Rev. Fluid Mech..

[24] Prieve D.C. (1982). Migration of a colloidal particle in a gradient of electrolyte concentration. Adv. Colloid Interface Sci..

[25] Zhang J., Wang Y., Wong T.I., Liu X., Zhou X., Liedberg B. (2015). Electrofocusing-enhanced localized surface plasmon resonance biosensors. Nanoscale.

[26] Prieve D.C., Malone S.M., Khair A.S., Stout R.F., Kanj M.Y. (2019). Diffusiophoresis of charged colloidal particles in the limit of very high salinity. Proc. Natl. Acad. Sci. USA.

[27] Kar A., Guha R., Dani N., Velegol D., Kumar M. (2014). Particle deposition on microporous membranes can be enhanced or reduced by salt gradients. Langmuir.

[28] Kar A., Chiang T.Y., Rivera I.O., Sen A., Velegol D. (2015). Enhanced transport into and out of dead-end pores. ACS Nano.

[29] Leaist D.G. (1989). The role of supporting electrolytes in protein diffusion. J. Phys. Chem..

[30] Gosting L.J. (1956). Measurement and interpretation of diffusion coefficients of proteins. Adv. Protein Chem..

[31] Albright J.G., Annunziata O., Miller D.G., Paduano L., Pearlstein A.J. (1999). Precision measurements of binary and multicomponent diffusion coefficients in protein solutions relevant to crystal growth: Lysozyme chloride in water and aqueous NaCl at ph 4.5 and 25 degrees c-perpendicular to. J. Am. Chem. Soc..

[32] McAfee M.S., Annunziata O. (2014). Effect of particle size on salt-induced diffusiophoresis compared to brownian mobility. Langmuir.

[33] McAfee M.S., Annunziata O. (2015). Effects of salting-in interactions on macromolecule diffusiophoresis and salt osmotic diffusion. Langmuir.

[34] Timasheff S.N. (2002). Protein-solvent preferential interactions, protein hydration, and the modulation of biochemical reactions by solvent components. Proc. Natl. Acad. Sci. USA.

[35] Arakawa T., Timasheff S.N. (1982). Preferential interactions of proteins with salts in concentrated solutions. Biochemistry.

[36] Jungwirth P., Cremer P.S. (2014). Beyond hofmeister. Nat. Chem..

[37] Annunziata O., Fahim A. (2020). A unified description of macroion diffusiophoresis, salt osmotic diffusion and collective diffusion coefficient. Int. J. Heat Mass Transf..

[38] Lightfoot E.N., Cussler E.L., Rettig R.L. (1962). Applicability of the stefan-maxwell equations to multicomponent diffusion in liquids. AIChE J..

[39] Krishna R. (2019). Diffusing uphill with james clerk maxwell and josef stefan. Chem. Eng. Sci..

[40] Lechlitner L.R., Annunziata O. (2018). Macromolecule diffusiophoresis induced by concentration gradients of aqueous osmolytes. Langmuir.

[41] Cruz Barrios E., Krause T.C., Annunziata O. (2022). Salt-induced diffusiophoresis of a nonionic micelle: Roles of salting out and proximity to surfactant cloud point. J. Mol. Liq..

[42] Leaist D.G. (1986). Diffusion of ionic micelles in salt-solutions–sodium dodecyl-sulfate + sodium-chloride + water. J. Colloid Interface Sci..

[43] Chelazzi D., Giorgi R., Baglioni P. (2018). Microemulsions, micelles, and functional gels: How colloids and soft matter preserve works of art. Angew. Chem. Int. Edit..

[44] Patist A., Kanicky J.R., Shukla P.K., Shah D.O. (2002). Importance of micellar kinetics in relation to technological processes. J. Colloid Interface Sci..

[45] Shah A., Shahzad S., Munir A., Nadagouda M.N., Khan G.S., Shams D.F., Dionysiou D.D., Rana U.A. (2016). Micelles as soil and water decontamination agents. Chem. Rev..

[46] La Sorella G., Strukul G., Scarso A. (2015). Recent advances in catalysis in micellar media. Green Chem..

[47] Lu Y., Zhang E.S., Yang J.H., Cao Z.Q. (2018). Strategies to improve micelle stability for drug delivery. Nano Res..

[48] Mangiapia G., D’Errico G., Simeone L., Irace C., Radulescu A., Di Pascale A., Colonna A., Montesarchio D., Paduano L. (2012). Ruthenium-based complex nanocarriers for cancer therapy. Biomaterials.

[49] Shin S., Um E., Sabass B., Ault J.T., Rahimi M., Warren P.B., Stone H.A. (2016). Size-dependent control of colloid transport via solute gradients in dead-end channels. Proc. Natl. Acad. Sci. USA.

[50] Rard J.A., Miller D.G. (1981). Isopiestic determination of the osmotic coefficients of aqueous Na_2_SO_4_, MgSO_4_, and Na_2_SO_4_-MgSO_4_ at 25-degrees-c. J. Chem. Eng. Data.

[51] Rard J.A., Miller D.G. (1979). Mutual diffusion-coefficients of na_2_so_4_-h2o and mgso_4_-h_2_o at 25 c from rayleigh interferometry. J. Solut. Chem..

[52] Wu J.Z., Liu F.H., Chen G., Wu X., Ma D.S., Liu Q.J., Xu S.J., Huang S.Z., Chen T., Zhang W. (2016). Effect of ionic strength on the interfacial forces between oil/brine/rock interfaces: A chemical force microscopy study. Energy Fuels.

[53] Strand S., Austad T., Puntervold T., Hognesen E.J., Olsen M., Barstad S.M.F. (2008). “Smart water” for oil recovery from fractured limestone: A preliminary study. Energy Fuels.

[54] Dharaiya N., Aswal V.K., Bahadur P. (2015). Characterization of triton x-100 and its oligomer (tyloxapol) micelles vis-à-vis solubilization of bisphenol a by spectral and scattering techniques. Colloids Surf. A Physicochem. Eng. Asp..

[55] Regev O., Zana R. (1999). Aggregation behavior of tyloxapol, a nonionic surfactant oligomer, in aqueous solution. J. Colloid Interface Sci..

[56] Schott H. (1998). Comparing the surface chemical properties and the effect of salts on the cloud point of a conventional nonionic surfactant, octoxynol 9 (triton x-100), and of its oligomer, tyloxapol (triton wr-1339). J. Colloid Interface Sci..

[57] Barrios E.C., Annunziata O. (2021). Determination of critical micelle concentration from the diffusion-driven dilution of micellar aqueous mixtures. Langmuir.

[58] Molina-Bolívar J.A., Aguiar J., Ruiz C.C. (2002). Growth and hydration of triton x-100 micelles in monovalent alkali salts: A light scattering study. J. Phys. Chem. B.

[59] Vergara A., Capuano F., Paduano L., Sartorio R. (2006). Lysozyme mutual diffusion in solutions crowded by poly(ethylene glycol). Macromolecules.

[60] Ribeiro A.C.F., Gomes J.C.S., Santos C.I.A.V., Lobo V.M.M., Esteso M.A., Leaist D.G. (2011). Ternary mutual diffusion coefficients of aqueous nicl2+ nacl and nicl2+ hcl solutions at 298.15 k. J. Chem. Eng. Data.

[61] Alexander N.P., Phillips R.J., Dungan S.R. (2019). Multicomponent diffusion in aqueous solutions of nonionic micelles and decane. Langmuir.

[62] Miller D.G., Vitagliano V., Sartorio R. (1986). Some comments on multicomponent diffusion–negative main term diffusion-coefficients, 2nd law constraints, solvent choices, and reference frame transformations. J. Phys. Chem..

[63] Zhang H.X., Annunziata O. (2009). Macromolecular hydration compared with preferential hydration and their role on macromolecule-osmolyte coupled diffusion. Phys. Chem. Chem. Phys..

[64] Pusey P.N., Tough R.J.A. (1985). Particle Interactions. Dynamic Light Scattering. Applications of Photon Correlation Spectroscopy.

[65] Rard J.A., Clegg S.L., Palmer D.A. (2000). Isopiestic determination of the osmotic coefficients of Na_2_SO_4_(aq) at 25 and 50c, and representation with ion-interaction (pitzer) and mole fraction thermodynamic models. J. Solut. Chem..

[66] Anderson C.F., Courtenay E.S., Record M.T. (2002). Thermodynamic expressions relating different types of preferential interaction coefficients in solutions containing two solute components. J. Phys. Chem. B.

[67] Weckstrom K., Papageorgiou A.C. (2007). Lower consolute boundaries of the nonionic surfactant c8e5 in aqueous alkali halide solutions: An approach to reproduce the effects of alkali halides on the cloud-point temperature. J. Colloid Interface Sci..

[68] McAfee M.S., Annunziata O. (2013). Cross-diffusion in a colloid-polymer aqueous system. Fluid Phase Equilib..

[69] Korosi A., Fabuss B.M. (2002). Viscosities of binary aqueous solutions of sodium chloride, potassium chloride, sodium sulfate, and magnesium sulfate at concentrations and temperatures of interest in desalination processes. J. Chem. Eng. Data.

[70] Corti M., Degiorgio V. (1981). Micellar properties and critical fluctuations in aqueous solutions of nonionic amphiphiles. J. Phys. Chem..

[71] Annunziata O., Buzatu D., Albright J.G. (2011). Effect of lysozyme proteins on the mutual-diffusion coefficient of sodium chloride in water. J. Chem. Eng. Data.

[72] Onsager L. (1931). Reciprocal relations in irreversible processes. I. Phys. Rev..

[73] Record M.T., Anderson C.F. (1995). Interpretation of preferential interaction coefficients of nonelectrolytes and of electrolyte ions in terms of a two-domain model. Biophys. J..

[74] Robson R.J., Dennis E.A. (1976). The size, shape, and hydration of nonionic surfactant micelles. Triton x-100. J. Phys. Chem..

[75] Perrin F. (1936). Mouvement brownien d’un ellipsoide (ii). Rotation libre et dépolarisation des fluorescences. Translation et diffusion de molécules ellipsoidales. J. Phys. Radium.

[76] Neurath H. (2002). The apparent shape of protein molecules. J. Am. Chem. Soc..

[77] Miller D.G., Albright J.G., Wakeham W.A., Nagashima A., Sengers J.V. (1991). Optical Methods. Measurement of the Transport Properties of Fluids: Experimental Thermodynamics.

[78] Annunziata O., Buzatu D., Albright J.G. (2005). Protein diffusion coefficients determined by macroscopic-gradient rayleigh interferometry and dynamic light scattering. Langmuir.

[79] Miller D.G. (1988). A method for obtaining multicomponent diffusion coefficients directly from rayleigh and gouy fringe position data. J. Phys. Chem..

[80] Zhang H., Annunziata O. (2008). Effect of macromolecular polydispersity on diffusion coefficients measured by rayleigh interferometry. J. Phys. Chem. B.

[81] Liu Z., Robinson J.T., Sun X.M., Dai H.J. (2008). Pegylated nanographene oxide for delivery of water-insoluble cancer drugs. J. Am. Chem. Soc..

[82] Kolate A., Baradia D., Patil S., Vhora I., Kore G., Misra A. (2014). Peg–a versatile conjugating ligand for drugs and drug delivery systems. J. Control. Release.

[83] Dong R.H., Hao J.C. (2010). Complex fluids of poly(oxyethylene) monoalkyl ether nonionic surfactants. Chem. Rev..

