# Supplementary material for: Diffusiophoresis of a Nonionic Micelle in Salt Gradients; Roles of Preferential Hydration and Salt-Induced Surfactant Aggregation
Source: Int J Mol Sci. 2022 Nov 8;23(22):13710. doi: 10.3390/ijms232213710 (PMC9696892; doi:10.3390/ijms232213710)
Supplement: Supplementary file 1 [file ijms-23-13710-s001.zip › ijms-1993940-supplementary.pdf]

Supplementary Data

Diffusiophoresis of a Nonionic Micelle in Salt Gradients: Roles of Preferential Hydration and Salt-Induced Surfactant Aggregation

Eliandreina Cruz Barrios, Kyra V. Penino and Onofrio Annunziata

S1. Thermodynamic and Transport Data

Our DLS diffusion coefficients (in the volume-fixed reference frame) are reported in Table S1.

Table S1. DLS diffusion coefficients of tyloxapol in aqueous $MgSO_4$ at 25 ºC.

$C_2 = 0\text{M}$

C_1/mM	$\mathcal{D}_1/10^{-12}$ m^2·s^{-1}
0.20	67.1±0.09
0.40	67.3±0.04
0.70	67.8±0.03
1.00	68.2±0.02

$C_2 = 0.10\text{M}$

C_1/mM	$\mathcal{D}_1/10^{-12}$ m^2·s^{-1}
0.20	66.6±0.06
0.40	65.7±0.02
0.70	64.9±0.05
1.00	63.9±0.03

$C_2 = 0.20\text{M}$

C_1/mM	$\mathcal{D}_1/10^{-12}$ m^2·s^{-1}
0.20	62.9±0.05
0.40	61.0±0.08
0.70	59.2±0.07
1.00	58.0±0.02

$C_2 = 0.30\text{M}$

C_1/mM	$\mathcal{D}_1/10^{-12}$ m^2·s^{-1}
0.20	54.5±0.04
0.40	52.7±0.01
0.70	49.7±0.04
1.00	47.4±0.02

$C_2 = 0.45\text{M}$

C_1/mM	$\mathcal{D}_1/10^{-12}$ m^2·s^{-1}
0.20	40.2±0.03
0.40	35.6±0.01
0.70	31.7±0.02
1.00	26.7±0.01

$C_2 = 0.60\text{M}$

C_1/mM	$\mathcal{D}_1/10^{-12}$ m^2·s^{-1}
0.20	26.2±0.02
0.40	21.9±0.01
0.70	18.0±0.01

$C_2 = 0.65M$

C_1/mM	$\mathcal{D}_1/10^{-12}$ m^2·s^{-1}
0.20	21.9±0.02
0.40	18.1±0.01
0.70	15.2±0.04
1.00	11.1±0.01

Thermodynamic and transport coefficients for the binary $MgSO_4$-water system at 25 ºC are reported in Table S2. Data of salt partial molar volume, $\bar{V}_2$, and thermodynamic factors, y_2, extracted from literature.[1,2] Mutual diffusion coefficients, $D_{2,V}$, of the binary salt-water system in the volume-fixed reference frame were measured in this work. Relative deviations of ternary values, D_{22}, from binary values: $r = D_{22}/D_{2,V} - 1$ are also included. Finally, relative viscosity coefficients, η_r, were taken from literature.[3]

Table S2. Thermodynamic and transport properties of aqueous $MgSO_4$ at 25 ºC.

C_2/M	$\bar{V}_2/\text{cm}^3 \cdot \text{mol}^{-1}$	y_2	$D_{2,V}/10^{-11}m^2 \cdot s^{-1}$	r /%	η_r
0.100	1.17	0.538	572	-1.5	1.072
0.300	4.65	0.498	496	-1.5	1.221
0.500	7.14	0.500	454	-2.6	1.399
0.650	8.73	0.518	429	-1.6	1.568

Micelle diffusiophoresis coefficients, $\widehat{D}_{12}$, and salt osmotic diffusion coefficients, $\widehat{D}_{21}$, are reported in Table S3. This table also reports micelle tracer diffusion coefficients, D_P, mobility ratios, α, and thermodynamic parameters, C_{21} and γ/m. The tracer diffusion coefficient of tyloxapol in the binary aqueous system was found to be 0.0690×10^{-12} m^2·s^{-1} by Rayleigh interferometry and 0.0668×10^{-12} m^2·s^{-1} by DLS. Thus, the DLS value is 3.3% lower. This discrepancy can be attributed to tyloxapol polydispersity. To calculate $\widehat{D}_{12}(C_2)$, we use the values of D_1 extracted from DLS measurements by linear extrapolation at $C_1 = 0$ and correct them by the factor of 1.033 to take into account this small discrepancy between interferometric and light scattering data. Value of D_1 at C_2 =0.65 M is obtained by linear interpolation of values of D_1 at C_2 =0.60 M and 0.69 M.

Table S3. Thermodynamic and transport parameters related to micelle diffusiophoresis.

C_2/M	$\widehat{D}_{12}$	$\widehat{D}_{21}$	$D_1/10^{-12}\text{m}^2 \cdot s^{-1}$	α	C_{21}	γ/m
0.10	0.52	1.23	70.3	0.1230	1.34	0.94
0.30	1.72	3.23	58.5	0.1177	3.47	2.28
0.50	4.02	5.76	45.2	0.0993	6.14	4.17
0.65	5.71	7.26	28.4	0.0658	7.56	5.00

S2. Effect of salt on surfactant aggregation. Consequences on diffusiophoresis

In this section, we explain the observed increase in micelle radius observed at high salt concentration. To explain experimental behavior, we assume that surfactant (S) unimers can reversibly make both spherical micelles (M) and larger aggregates (A) with well-defined aggregation numbers. In our model, the observed micelle radius is a weighted average between micelle and aggregate radius. These equilibria can be described by considering the following reversible reactions:

$$m\text{S} \rightleftharpoons \text{M} \qquad am\text{S} \rightleftharpoons \text{A}$$

where m is the micelle aggregation number and a is the molecular-weight ratio of aggregate to micelle. We use the symbols "1" and "2" to indicate the surfactant and salt components, respectively. The total surfactant concentration is

$$C_1 = C_\text{S} + mC_\text{M} + a\,mC_\text{A} \tag{S1}$$

where C_S, C_M and C_A are the concentrations of free surfactant, micelles, and aggregates, respectively. In principle, the fraction of aggregates and micelles become zero in the limit of $C_1 \to 0$ as all surfactant should occur as free unimers at infinite dilution. Furthermore, dilution also favors micelles with respect to aggregates because aggregate formation requires a larger number of unimers compared to micelles. However, surfactant concentrations in which disaggregation is favored may be sufficiently low that cannot be accessed experimentally. For instance, tyloxapol critical micelle concentration is sufficiently low that we can neglect C_S in Eq. S1. Furthermore, extrapolation to $C_1 \to 0$ of experimental data will practically yield physicochemical quantities that relate to micelles not free surfactant. A similar argument will be proposed below for aggregates with respect to smaller micelles.

If we neglect free surfactant in Eq. S1, we can write:

$$C_1 \cong mC_\text{M} + a\,mC_\text{A} \tag{S2}$$

and the fraction of aggregated surfactant is

$$X_{\rm A} \equiv \frac{amC_{\rm A}}{C_1} \cong \frac{amC_{\rm A}}{mC_{\rm M}+amC_{\rm A}} \qquad \text{(S3)}$$

with $1-X_{\rm A}$ being the fraction of micellar surfactant. In our model, the observed behavior of Stokes radius, $R_{\rm P}(C_2)$, is caused by an increase in $X_{\rm A}$ with salt concentration. Our goal is to determine a mathematical expression for $X_{\rm A}$.

Micelle-aggregate chemical equilibrium may be described by

$$\frac{C_{\rm A}}{C_{\rm M}^{\ a}} = \left(\frac{C_1^*}{m}\right)^{1-a} \qquad \text{(S4)}$$

where $(C_1^*/m)^{1-a}$ is the equilibrium constant, rewritten so that C_1^* is a critical surfactant concentration above which aggregates become favored with respect to micelles. We can then rewrite Eq. S4 using the fraction of aggregated surfactant given by Eq. S3:

$$\frac{X_{\rm A}}{1-X_{\rm A}} = a\left(\frac{mC_{\rm M}}{C_1^*}\right)^{a-1} \qquad \text{(S5)}$$

To introduce the effect of salt concentration on micelle-aggregate chemical equilibrium, we assume that C_1^* depends on salt concentration, C_2. Since salt promotes formation of aggregates at high salt concentration, C_1^* must decrease as salt concentration increases. This salting-out effect can be described by assuming that $\ln C_1^*$ linearly decreases as C_2 increases according to:

$$\ln C_1^* = \ln C_1^{0*} - K_2' C_2 \qquad \text{(S6)}$$

where C_1^{0*} is the value of C_1^* in the absence of salt and the coefficient, K_2', is a salting-out constant. The physical meaning of K_2' can be explained using the preferential-hydration model. Within model framework, K_2' is directly proportional to $N_{\rm W}^{\rm (M)} - N_{\rm W}^{\rm (A)}$, where $N_{\rm W}^{\rm (M)}$ and $N_{\rm W}^{\rm (A)}$ are water excesses per surfactant unit in the micellar and aggregate state, respectively. The positive parameters, $N_{\rm W}^{\rm (M)}$ and $N_{\rm W}^{\rm (A)}$, characterize how surfactant chemical potential in micelle and aggregate states increases with salt concentration. If the surfactant chemical potential in the micelle state increases more rapidly than that in the aggregate state ($N_{\rm W}^{\rm (M)} > N_{\rm W}^{\rm (A)}$) then $K_2' > 0$. This implies that there exists a salt concentration above which aggregates become more stable than micelles.

Based on Eq. S6, the ratio, $mC_{\rm M}/C_1^*$ in Eq. S5, can be rewritten in the following way:

$$\frac{mC_{\rm M}}{C_1^*} = \frac{mC_{\rm M}}{C_1}\frac{C_1}{C_1^*} = (1-X_{\rm A})\frac{C_1}{C_1^{0*}} e^{K_2' C_2} \qquad \text{(S7)}$$

To ensure that $mC_{\rm M}/C_1^*$ and $X_{\rm A}$ in Eqs. S6,7 do not vanish in the limit of $C_1 \to 0$, we should interpret C_1^{0*} as infinitely small. From experimental point of view, this corresponds to C_1^{0*} being low compared to experimental surfactant concentrations. Thus, the factor $(C_1/C_1^{0*})\ e^{K_2' C_2}$ in Eq. S7 will also not vanish in the limit of $C_1 \to 0$. We expect that C_1/C_1^{0*} is a small fraction of 1 as aggregates are negligible compared to micelles in water. However, as salt concentration increases, the factor $(C_1/C_1^{0*})\ e^{K_2' C_2}$ increases thereby making the fraction of aggregates no longer negligible. It is convenient to introduce a salt concentration C_2^* such that $(C_1/C_1^{0*})\ e^{K_2' C_2^*} = 1$. We can then rewrite Eq. S7 in the following way:

$$\frac{mC_{\mathrm{M}}}{C_1^*} = (1 - X_{\mathrm{A}})\, e^{K_2'(C_2 - C_2^*)} \tag{S8}$$

This expression of mC_{M} / C_1^* can be then inserted into Eq. S5 to finally obtain:

$$X_{\mathrm{A}} = a\,(1 - X_{\mathrm{A}})^a\, e^{K_2(C_2 - C_2^*)} \tag{S9}$$

which is the same as Eq. 11 in the manuscript with $K_2 \equiv (a-1)K_2'$. If the parameters a, K_2 and C_2^* are known, Eq. S9 can be numerically solved for X_{A} using Newtown's method starting from the seed value of $X_{\mathrm{A}} = 0$.

We now turn our attention to the normalized Stokes' radius, $R_{\mathrm{P}}(C_2)/R_{\mathrm{P}}(0)$, and reduced diffusiophoresis coefficient, $\hat{D}_{12}(C_2)$, and their relation with X_{A}. To describe the effect of aggregation on surfactant diffusion, we assume simple Fick's first law for micelle and aggregate:

$$J_{\mathrm{M}} = -D_{\mathrm{M}} \nabla C_{\mathrm{M}} \tag{S10}$$

$$J_{\mathrm{A}} = -D_{\mathrm{A}} \nabla C_{\mathrm{A}} \tag{S11}$$

where J_{M} and J_{A} are micelle and aggregate fluxes, and D_{M} and D_{A} the corresponding diffusion coefficients. According to mass balance (see Eq. S2), the total surfactant flux is given by

$$\frac{J_1}{m} = J_{\mathrm{M}} + a\, J_{\mathrm{A}} = -D_{\mathrm{M}} \nabla C_{\mathrm{M}} - a\, D_{\mathrm{A}} \nabla C_{\mathrm{A}} \tag{S12}$$

Assuming rapid equilibrium, the concentration gradients ∇C_{M} and ∇C_{A} can be related to ∇C_1 and ∇C_2 by

$$\nabla C_{\mathrm{M}} = \left(\frac{\partial C_{\mathrm{M}}}{\partial C_1}\right)_{C_2} \nabla C_1 + \left(\frac{\partial C_{\mathrm{M}}}{\partial C_2}\right)_{C_1} \nabla C_2 \tag{S13}$$

$$\nabla C_{\mathrm{A}} = \left(\frac{\partial C_{\mathrm{A}}}{\partial C_1}\right)_{C_2} \nabla C_1 + \left(\frac{\partial C_{\mathrm{A}}}{\partial C_2}\right)_{C_1} \nabla C_2 \tag{S14}$$

Accordingly, Eq. S12 becomes:

$$J_1 = -D_{11} \nabla C_1 - D_{12} \nabla C_2 \tag{S15}$$

where

$$D_{11} = m\left(\frac{\partial C_{\mathrm{M}}}{\partial C_1}\right)_{C_2} D_{\mathrm{M}} + ma\left(\frac{\partial C_{\mathrm{A}}}{\partial C_1}\right)_{C_2} D_{\mathrm{A}} \tag{S16}$$

$$D_{12} = m\left(\frac{\partial C_{\mathrm{M}}}{\partial C_2}\right)_{C_1} D_{\mathrm{M}} + ma\left(\frac{\partial C_{\mathrm{A}}}{\partial C_2}\right)_{C_1} D_{\mathrm{A}} \tag{S17}$$

The four partial derivatives appearing in Eqs. S16,17 can be expressed as functions of X_{A}. Since $aC_{\mathrm{A}} = (C_1/m)\,X_{\mathrm{A}}$ and $C_{\mathrm{M}} = (C_1/m)(1 - X_{\mathrm{A}})$, we can first write:

$$m\left(\frac{\partial C_{\mathrm{M}}}{\partial C_1}\right)_{C_2} = (1-X_{\mathrm{A}}) - C_1\left(\frac{\partial X_{\mathrm{A}}}{\partial C_1}\right)_{C_2} \tag{S18}$$

$$ma\left(\frac{\partial C_{\mathrm{A}}}{\partial C_1}\right)_{C_2} = X_{\mathrm{A}} + C_1\left(\frac{\partial X_{\mathrm{A}}}{\partial C_1}\right)_{C_2} \tag{S19}$$

$$m\left(\frac{\partial C_{\mathrm{M}}}{\partial C_2}\right)_{C_1} = -C_1\left(\frac{\partial X_{\mathrm{A}}}{\partial C_2}\right)_{C_1} \tag{S20}$$

$$ma\left(\frac{\partial C_{\mathrm{A}}}{\partial C_2}\right)_{C_1} = C_1\left(\frac{\partial X_{\mathrm{A}}}{\partial C_2}\right)_{C_1} \tag{S21}$$

We can then deduce the expressions of $(\partial X_{\mathrm{A}} / \partial C_1)_{C_2}$ and $(\partial X_{\mathrm{A}} / \partial C_2)_{C_1}$ from Eq. S9 reported below:

$$\left(\frac{\partial X_{\mathrm{A}}}{\partial C_1}\right)_{C_2} = -\frac{aX_{\mathrm{A}}}{1-X_{\mathrm{A}}}\left(\frac{\partial X_{\mathrm{A}}}{\partial C_1}\right)_{C_2} - K_2 X_{\mathrm{A}} \frac{dC_2^*}{dC_1} \tag{S22}$$

$$\left(\frac{\partial X_{\mathrm{A}}}{\partial C_2}\right)_{C_1} = -\frac{a X_{\mathrm{A}}}{1-X_{\mathrm{A}}}\left(\frac{\partial X_{\mathrm{A}}}{\partial C_2}\right)_{C_1} + K_2 X_{\mathrm{A}} \tag{S23}$$

To determine dC_2^* / dC_1 in Eq. S22, we observe that C_2^* is defined such that $K_2' C_2^* = -\ln(C_1 / C_1^{0*})$. This implies that $K_2 (dC_2^* / dC_1) = -(a-1)(1/C_1)$. Thus, the final expressions of $(\partial X_{\mathrm{A}} / \partial C_1)_{C_2}$ and $(\partial X_{\mathrm{A}} / \partial C_2)_{C_1}$ are

$$\left(\frac{\partial X_{\mathrm{A}}}{\partial C_1}\right)_{C_2} = \frac{1}{C_1}\frac{(a-1)\,(1-X_{\mathrm{A}})X_{\mathrm{A}}}{1+(a-1)X_{\mathrm{A}}} \tag{S24}$$

$$\left(\frac{\partial X_{\mathrm{A}}}{\partial C_2}\right)_{C_1} = K_2\frac{(1-X_{\mathrm{A}})X_{\mathrm{A}}}{1+(a-1)X_{\mathrm{A}}} \tag{S25}$$

Substitution of Eqs. S24,25 into Eqs. S18-21 allows us to rewrite Eqs. S16,17 in the following way:

$$D_{11} = \frac{(1-X_{\mathrm{A}})D_{\mathrm{M}} + aX_{\mathrm{A}}D_{\mathrm{A}}}{(1-X_{\mathrm{A}}) + aX_{\mathrm{A}}} \tag{S28}$$

$$D_{12} = -C_1\frac{K_2\nu_2\,(1-X_{\mathrm{A}})X_{\mathrm{A}}}{(1-X_{\mathrm{A}}) + a\,X_{\mathrm{A}}}(D_{\mathrm{M}} - D_{\mathrm{A}}) \tag{S29}$$

In Eq. S28, we obtain $D_{11} \cong D_{\mathrm{M}}$ if $X_{\mathrm{A}} << 1$ as expected. This condition is approximately achieved in the absence of salt ($C_2 = 0$). Since the observed Stokes radius, $R_{\mathrm{P}}(C_2)$, is inversely proportional to D_{11}, we finally obtain Eq. 11 in the manuscript:

$$\frac{R_{\mathrm{P}}}{R_{\mathrm{P}}^0} = \frac{1-X_{\mathrm{A}} + a\,X_{\mathrm{A}}}{1-X_{\mathrm{A}} + a\,X_{\mathrm{A}}\,\alpha_a} \tag{S30}$$

where $R_{\mathrm{P}}^0 \equiv R_{\mathrm{P}}(0)$ and $\alpha_a \equiv D_{\mathrm{A}} / D_{\mathrm{M}}$ is a mobility ratio. According to Eq. S30, R_{P} increases with X_{A} when $\alpha_a < 1$. It is interesting to observe that the same expression of R_{P} can be obtained in dynamic light

scattering by assuming that that micelles and aggregates are not in chemical equilibrium. Indeed, $a\,X_\mathrm{A}$ represents the light-scattering weight of the aggregates.

To obtain the expression of the reduced diffusiophoresis coefficient, $\hat{D}_{12}$, we combine Eq. S29 with Eq. S28 as shown below:

$$\hat{D}_{12} = \frac{D_{12}C_2}{\nu_2 D_{11} C_1} = -\frac{(1-X_\mathrm{A})X_\mathrm{A}}{1-X_\mathrm{A}+a\,X_\mathrm{A}\alpha_a}\frac{(1-\alpha_a)K_2C_2}{\nu_2} \tag{S31}$$

which is Eq. 13 in the manuscript.

To calculate $\hat{D}_{12}(C_2)$, we need to know the values of a, α_a, K_2 and X_A, with X_A obtained from a, K_2 and C_2^* using Eq. S9. To reduce number of parameters, we set the value of a equal to three representative values: $a = 10$, 20 and 100 and calculate α_a from a by assuming that micelles are spheres and aggregates are prolate ellipsoids with minor semiaxis equal to micelle radius, R_M, and major semiaxis, $R_\mathrm{A} > R_\mathrm{M}$. The diffusion-coefficient ratio can then be written as

$$\alpha_a = \frac{D_\mathrm{A}}{D_\mathrm{M}} = \frac{R_\mathrm{M}}{(R_\mathrm{A}R_\mathrm{M}{}^2)^{1/3}}\varphi \tag{S32}$$

where φ is the Perrin shape factor.[4,5] For a prolate ellipsoid, we can write:

$$\varphi = \frac{(R_\mathrm{M}/R_\mathrm{A})^{2/3}}{\sqrt{1-(R_\mathrm{M}/R_\mathrm{A})^2}}\ln\frac{1+\sqrt{1-(R_\mathrm{M}/R_\mathrm{A})^2}}{(R_\mathrm{M}/R_\mathrm{A})} \tag{S33}$$

Assuming micelle and aggregate share the same density, the ratio of aggregate-to-micelle volume $R_\mathrm{A}R_\mathrm{M}{}^2/R_\mathrm{M}{}^3 = R_\mathrm{A}/R_\mathrm{M}$ is equal to the ratio of aggregate-to-micelle aggregation numbers, a. This leads to

$$\alpha_a = \frac{\ln\left(a+\sqrt{a^2-1}\right)}{\sqrt{a^2-1}} \tag{S34}$$

Thus, for a given value of a, α_a is directly obtained using Eq. S34. We then use experimental data of $R_\mathrm{P}(C_2)$ to extract K_2 and C_2^*. Specifically, we combine Eqs. S9,S30 to write:

$$\frac{R_\mathrm{P}}{R_\mathrm{P}^0} = \frac{1+a(a-1)\,e^{K_2(C_2-C_2^*)}(1-X_\mathrm{A})^a}{1+a(a\;\alpha_a-1)e^{K_2(C_2-C_2^*)}(1-X_\mathrm{A})^a} \tag{S35}$$

According to Eq. S35, $R_\mathrm{P}/R_\mathrm{P}^0$ depends on two variables, C_2 and X_A, while K_2 and C_2^* being the two parameters to be determined. However, the set of values of X_A to be used on the right side of Eq. S9 is initially unknown as they depend on K_2 and C_2^*. We therefore choose two approximate values of K_2 and C_2^* and calculate X_A at each experimental C_2 from Eq. S9. Specifically, we numerically solve Eq. S9 using Newton's method starting with $X_\mathrm{A}^{(0)} = 0$:

$$X_\mathrm{A}^{(i)} = X_\mathrm{A}^{(i-1)} - \frac{X_\mathrm{A}^{(i-1)} - a\,(1-X_\mathrm{A}^{(i-1)})^a\,e^{K_2(C_2-C_2^*)}}{1+a^2\,(1-X_\mathrm{A}^{(i-1)})^{a-1}\,e^{K_2(C_2-C_2^*)}} \qquad \text{with} \qquad i = 1,2,3,... \tag{S36}$$

The method of least squares based on Eq. S35 is then applied to $R_P(C_2, X_A)$ data and new values of K_2 and C_2^* are extracted. These are then used to recalculate the set of values of X_A using Eq. S36. This procedure is repeated until values of K_2 and C_2^* remain the same within their statistical uncertainties. Our results are reported in Table S6 below.

Table S6. Parameters extracted by applying the method least squares based on Eq. S35.

a	α_a	Na_2SO_4	K_2/M^{-1}	C_2^*/M	$MgSO_4$	K_2/M^{-1}	C_2^*/M
10	0.3008		19±3	0.72±0.02		16±3	0.89±0.03
20	0.1846		15±2	0.91±0.03		13±3	1.08±0.08
100	0.0530		12±2	1.3±0.1		11±3	1.5±0.2

References

(1) Rard, J. A.; Miller, D. G. mutual diffusion-coefficients of Na2SO4-h2o and MgSO4-h2o at 25-degrees-c from rayleigh interferometry. *Journal of Solution Chemistry* **1979**, *8* (10), 755-766. DOI: 10.1007/bf00648779.

(2) Rard, J. A.; Miller, D. G. Isopiestic determination of the osmotic coefficients of aqueous Na2SO4, MgSO4, and Na2SO4-MgSO4 at 25 C. *J. Chem. Eng. Data* **1981**, *26* (1), 33-38. DOI: 10.1021/je00023a013.

(3) Korosi, A.; Fabuss, B. M. Viscosities of binary aqueous solutions of sodium chloride, potassium chloride, sodium sulfate, and magnesium sulfate at concentrations and temperatures of interest in desalination processes. *Journal of Chemical & Engineering Data* **2002**, *13* (4), 548-552. DOI: 10.1021/je60039a031.

(4) Neurath, H. The Apparent Shape of Protein Molecules. *J. Am. Chem. Soc.* **2002**, *61* (7), 1841-1844. DOI: 10.1021/ja01876a060.

(5) Perrin, F. Mouvement Brownien d'un ellipsoide (II). Rotation libre et dépolarisation des fluorescences. Translation et diffusion de molécules ellipsoidales. *J. Phys. Radium* **1936**, *7* (1), 1-11, 10.1051/jphysrad:01936007010100.
